# Fermentation with Tea Residues Enhances Antioxidant Activities and Polyphenol Contents in Kombucha Beverages

**DOI:** 10.3390/antiox11010155

**Published:** 2022-01-14

**Authors:** Dan-Dan Zhou, Adila Saimaiti, Min Luo, Si-Yu Huang, Ruo-Gu Xiong, Ao Shang, Ren-You Gan, Hua-Bin Li

**Affiliations:** 1 Guangdong Provincial Key Laboratory of Food, Nutrition and Health, Department of Nutrition, School of Public Health, Sun Yat-sen University, Guangzhou 510080, China; zhoudd6@mail2.sysu.edu.cn (D.-D.Z.); saimaiti@mail2.sysu.edu.cn (A.S.); luom65@mail2.sysu.edu.cn (M.L.); huangsy9@mail2.sysu.edu.cn (S.-Y.H.); xiongrg@mail2.sysu.edu.cn (R.-G.X.); shangao@connect.hku.hk (A.S.); 2 School of Chinese Medicine, Li Ka Shing Faculty of Medicine, The University of Hong Kong, Hong Kong 999077, China; 3 Research Center for Plants and Human Health, Institute of Urban Agriculture, Chinese Academy of Agricultural Sciences, Chengdu 610213, China

**Keywords:** tea, kombucha beverage, tea residue, fermentation, antioxidant activity, polyphenols

## Abstract

Kombucha is a popular beverage with various bioactivities (such as antioxidant activity), which can be attributed to its abundant bioactive compounds, especially polyphenols. Kombucha is conventionally prepared by fermentation of a sugared black tea infusion without tea residue. In this study, the effects of black tea residue and green tea residue on kombucha were studied, and its antioxidant activities, total phenolic contents, as well as concentrations of polyphenols at different fermentation stages were evaluated using ferric-reducing antioxidant power, Trolox equivalent antioxidant capacity, Folin-Ciocalteu method and high-performance liquid chromatography with a photodiode array detector. The results showed that fermentation with tea residue could markedly increase antioxidant activities (maximum 3.25 times) as well as polyphenolic concentrations (5.68 times) of kombucha. In addition, green tea residue showed a stronger effect than black tea residue. Overall, it is interesting to find that fermentation with tea residues could be a better strategy to produce polyphenol-rich kombucha beverages.

## 1. Introduction

Tea (*Camellia sinensis*) is a widely consumed beverage with a long drinking history [1,2]. It can be divided into six main categories according to its processing and fermentation, that is, green tea (unfermented), white tea (slightly fermented), yellow tea (slightly fermented), oolong tea (semi-fermented), black tea (completely fermented) and dark tea (post-fermented) [3,4]. Kombucha is traditionally prepared by the fermentation of a sugared black tea infusion with tea fungus (a symbiotic culture of lactic acid bacteria, acetic acid bacteria and yeasts) [5,6]. The tea fungus usually contains many different microorganisms and is also affected by the microorganisms in the cultural environment, which will affect the composition of kombucha beverages. Kombucha is a popular beverage and possesses a variety of bioactive compounds, including polyphenols, vitamins, minerals, lipids, proteins and diverse metabolic products of yeasts and bacteria. The presence of these bioactive compounds, especially polyphenols, confers kombucha multiple bioactivities, such as antioxidant, immunomodulatory, antihypertensive, hypoglycemic, cholesterol-lowering, hepatoprotective, antiproliferative, and antimicrobial activities [7,8].

Although kombucha could be prepared from different types of teas (e.g., black and green teas) and some non-*Camellia sinensis* plants, it is commonly produced by the fermentation of a sugared black tea or green tea infusion without tea residue [9,10,11]. According to our previous studies as well as the experiences of people brewing tea leaves, it is very difficult to extract most of the bioactive compounds in tea leaves by a single boiling water extraction [4,12]. Therefore, it is hypothesized that the fermentation together with tea residue can markedly increase the antioxidant activities and polyphenol contents of kombucha. In this research, the effects of black tea residue and green tea residue on kombucha are studied at different fermentation stages, including antioxidant activities, total phenolic contents as well as concentrations of several polyphenols. The results support our hypothesis, and the fermentation with tea residue could be a better strategy to produce kombucha rich in antioxidants, such as polyphenols.

## 2. Materials and Methods

### 2.1. Chemicals and Materials

Sucrose was obtained from the Macklin Chemical Factory (Shanghai, China). Gallic acid, Folin-Ciocalteu’s phenol reagent, 2,2′-azinobis (3-ethylbenothiazoline-6-sulfonic acid) diammonium salt (ABTS), 6-hydroxy-2,5,7,8-tetramethylchromane-2-carboxylic acid (Trolox) and 2,4,6-tri(2-pyridyl)-S-triazine (TPTZ) were bought from Sigma-Aldrich (St. Louis, MO, USA). Ethanol, acetic acid, hydrochloric acid, potassium persulfate, iron(II) sulfate heptahydrate, iron(III) chloride hexahydrate and sodium acetate were obtained from Tianjin Chemical Factory (Tianjin, China). Sodium carbonate was bought from Shanghai Yuanye Biological Technology Co., Ltd. (Shanghai, China). Methanol and formic acid were purchased from Macklin Chemical Factory (Shanghai, China). The standard chemicals, including catechin (C), catechin gallate (CG), gallocatechin (GC), epicatechin (EC), epicatechin gallate (ECG), epigallocatechin (EGC), gallocatechin gallate (GCG), epigallocatechin gallate (EGCG), astragalin, caffeine, chlorogenic acid, ellagic acid, gallic acid, kaempferol, myricetin, quercetin, quercitrin and theaflavin, were bought from Derick Biotechnology Co., Ltd. (Chengdu, China). The distilled water was used.

Black tea (Dianhong tea) was obtained from Yunnan Dianhong Group Co., Ltd. (Kunming, China), and green tea (Dianqing tea) was bought from Yunnan Dianqing Tea Co., Ltd. (Kunming, China). The kombucha starter culture, including tea fungus, fermented broth, cellulosic layer and a teabag with 5 g black tea, was acquired from Shandong Ruyun Edible Fungus Planting Co., Ltd. (Liaocheng, China), and stored in a refrigerator at 4 °C.

### 2.2. Activation of Kombucha Starter Culture

Activation of the Kombucha starter culture was carried out according to the instruction of the manufacturer. The teabag, 100 g sugar and 1 L boiling water were added into a sterilized glass jar and mixed. After cooling to room temperature (25 °C), the teabag was removed, and tea fungus, fermented broth and cellulosic layer were added. The mixture was fermented for 14 days in a clean and dark place at room temperature for the subsequent inoculation.

### 2.3. Preparation of Kombucha with or without Tea Residue

The preparation of kombucha was carried out according to the literature with minor modifications [13,14,15]. The 300 mL distilled water was added to a 500 mL conical flask and heated to boiling. Then, 30 g sucrose was added and completely dissolved. Finally, 3 g tea was added to the mixture for 5 min, and then cooled to room temperature. The mixture was filtered through a sieve to collect the infusion for fermentation without residue. For fermentation with tea residue, the mixture was not filtered. Then, 30 mL activated kombucha starter culture was added into a conical flask containing 300 mL sugared tea infusion with or without tea residue. The conical flask was carefully covered with a clean cloth and fastened appropriately. The fermentation process was performed for 15 days in a clean and dark place at room temperature. The sample was collected on days 0, 3, 6, 9, 12 and 15, and filtrated through a 0.45 µm membrane for the determination of antioxidant activity and polyphenol contents. The experiments were repeated three times, and the results were expressed as mean ± standard deviation.

### 2.4. Measurement of Antioxidant Capacity and Total Phenolic Content

The antioxidant activity, including ferric-reducing antioxidant power (FRAP) and Trolox equivalent antioxidant capacity (TEAC), and total phenolic content (TPC) of samples were measured based on the published literature [9,16,17,18].

For the FRAP assay, sodium acetate-acetic acid buffer (300 mmol/L), TPTZ solution (10 mmol/L), and ferric chloride solution (20 mmol/L) were freshly mixed at a volume ratio of 10:1:1 for preparing FRAP reagent, which was placed in a water bath at 37 °C for follow-up experiments. Then, the properly diluted sample of 100 µL was added to 3 mL of FRAP reagent and reacted at room temperature for 4 min. Finally, the absorbance was recorded at 593 nm. FeSO_4_ was used as the standard, and the FRAP values were expressed as mmol Fe(II)/L.

For the TEAC assay, potassium persulfate solution (2.45 mmol/L) and ABTS solution (7 mmol/L) were mixed at a volume ratio of 1:1 for preparing ABTS^∙+^ stock solution, which could be used after 16 h of incubation in the dark, and was effective within 48 h. The stock solution was diluted to an absorbance of 0.71 ± 0.05 at 734 nm, and the obtained dilution ratio was used to prepare the reaction solution. Then, 100 µL diluted sample solution was mixed with a 3.8 mL reaction solution, and the mixture reacted for 6 min in the dark at room temperature. Subsequently, the absorbance of the sample was determined at 734 nm. Trolox was used as the standard, and the results were expressed as mmol Trolox/L.

The TPC of the samples was determined using the Folin-Ciocalteu method. The 0.50 mL of diluted sample solution was added to 2.5 mL of the Folin-Ciocalteu reagent (0.2 mol/L) and reacted for 4 min in the dark at room temperature. Then, 2 mL of saturated sodium carbonate solution (75 g/L) was added and then incubated in the dark for 2 h at room temperature. Finally, the absorbance was evaluated at 760 nm. Gallic acid was used as the standard, and the TPC value was shown as mg of gallic acid equivalent (GAE)/L.

### 2.5. Determination of Phenolic Compounds and Caffeine by HPLC-PDAD

The contents of phenolic compounds and caffeine in kombucha beverages were qualitatively and quantitatively determined by HPLC coupled with a photodiode array detector (PDA) (Waters, Milford, MA, USA) according to the literature [19,20,21,22,23]. An Agilent Zorbax Eclipse XDB-C18 column (4.6 × 250 mm, 5 µm, Santa Clara, CA, USA) was used for the separation. The column temperature was set at 35 °C with a flow rate of 0.8 mL/min. The mobile phases A and B were methanol and 0.1% formic (*v*/*v*), respectively. The gradient elution procedure was set as follows: 0–10 min, 2–17% A; 10–15 min, 17–19% A; 15–20 min; 19–22% A; 20–40 min, 22–47% A; 40–50 min, 47–50% A; 50–60 min, 50–58% A; 60–70 min, 58–2% A; and 70–75 min, 2% A. Phenolic compounds and caffeine in kombucha beverages were identified via comparing their retention time and ultraviolet-visible spectra with those of standard compounds, and the peak area under the maximal absorbance wavelength was used for quantification, with their contents expressed as mg/L.

### 2.6. Sensory Analysis

The sensory panel analysis of kombucha beverages was performed according to the literature [15,24]. The kombucha beverages were scored by seven graduate students and one professor (23–58 years old) from the Department of Nutrition, School of Public Health, Sun Yat-Sen University. The scores for color, odour, taste, sourness, and overall acceptability were given by each person, and a scoring range of 1–9 was used, including extreme disliking (1 point), great disliking (2 points), moderate disliking (3 points), slight disliking (4 points), neither liking nordisliking (5 points), slight liking (6 points), moderate liking (7 points), great liking (8 points), and extreme liking (9 points).

### 2.7. Statistical Analysis

All experimental results were expressed as mean ± standard deviation. All data analysis was carried out by Excel 2010 (Microsoft, Washington, DC, USA), and SPSS 26.0 statistics software (IBM Corp., Armonk, NY, USA). R software (version 4.1.2 (Bird Hippie), R Foundation for Statistical Computing, Vienna, Austria) is used for the correlation analysis. The one-way analysis of variance (ANOVA) was applied to analyze the statistical significance. Statistical significance was defined at *p* < 0.05.

## 3. Results and Discussion

**Figure 1:**
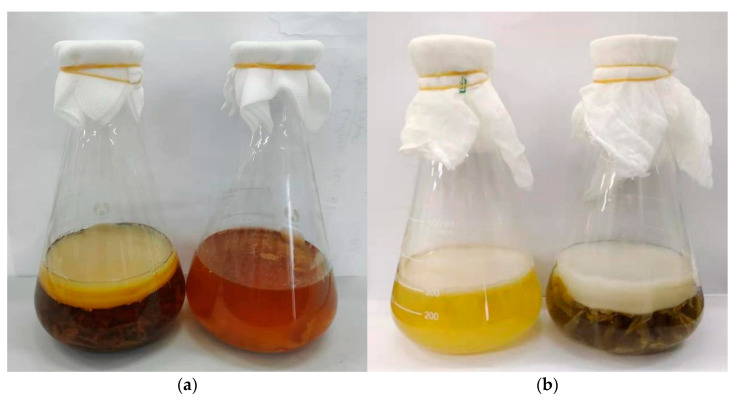
The appearance of kombucha produced by fermentation with or without tea residue. (**a**) kombucha from black tea with or without tea residue; (**b**) kombucha from green tea with or without tea residue.

Kombucha has shown various beneficial effects, and antioxidant activity is one of its most important bioactivities [25]. In addition, its antioxidant activity, as well as polyphenols, could also be the basis of its other beneficial effects. Therefore, this study focused on the effects of tea residue on the antioxidant capacities as well as the polyphenol concentrations of kombucha. The appearance of kombucha produced by fermentation with or without tea residue is shown in Figure 1.

### 3.1. FRAP Values of Kombucha with or without Tea Residue

**Figure 2:**
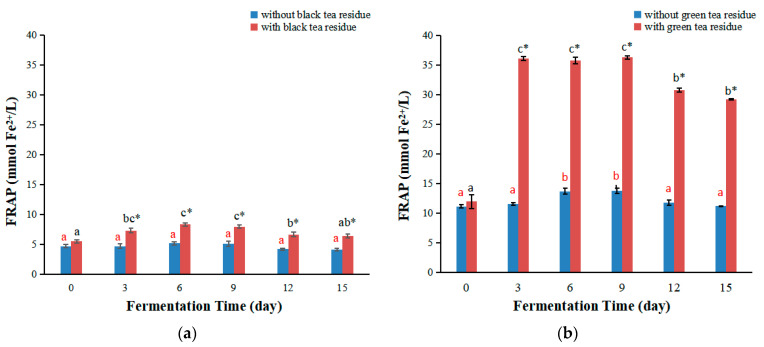
Changes of FRAP values of kombucha produced by fermentation with or without tea residue at different fermentation stages. (**a**) FRAP values of kombucha from black tea; (**b**) FRAP values of kombucha from green tea. Different letters illustrate significant differences (*p* < 0.05) for the same kombucha beverage at different fermentation times, and the same letter represents no significant difference (*p* > 0.05). Different colors of letters represent different kombucha fermentation with tea residue or without tea residue. * Indicates significant difference (*p* < 0.05) between kombucha fermentation with tea residue and kombucha fermentation without tea residue at the same fermentation time.

The FRAP assay estimates the ability to reduce ferric(III) ions to ferrous(II) ions, and is a simple, inexpensive, reproducible and commonly employed method for evaluating antioxidant capacity [26]. The FRAP values of kombucha from black tea are shown in Figure 2a. Generally, the FRAP values of kombucha firstly increased with the increase of fermentation time and reached the maximum on day six, then decreased. The change trend of FRAP values of kombucha from black tea without tea residue was similar to that reported in the literature [14,27]. Remarkably, the FRAP values of kombucha with black tea residues were significantly higher than those without tea residues, with 1.6 folds higher on day six (Figure 2a).

The FRAP values of kombucha from green tea are shown in Figure 2b, which firstly increased with the extension of fermentation time, and then decreased. The maximum values of kombucha with or without tea residue were obtained on day three and day six, respectively. The change trend of FRAP values of kombucha from green tea without tea residue was also similar to that reported in the literature [14,27]. Furthermore, the FRAP values of kombucha with green tea residue were markedly higher than those without tea residue, with 3.13 and 2.62 folds higher on day three and day six, respectively (Figure 2b).

In brief, fermentation with tea residue significantly enhances the FRAP values of kombucha beverages, and green tea residue showed a stronger effect than black tea residue. These differences could be caused by many factors. The time for the extraction of bioactive compounds from tea leaves could be an influencing factor. In the literature, the extraction time was usually 5 min with boiling water [25,27,28]. In this study, tea leaves were firstly boiled for 5 min and then cooled to room temperature, which cost about 30 min. In fact, it is very difficult to extract all bioactive compounds from tea leaves with boiling water by a single extraction according to the experiences of people brewing tea leaves (with many times dip using boiling water) as well as our previous studies [4,12]. Therefore, some of the bioactive compounds would still be left in tea leaves, which could be dissolved into the fermentation broth, especially under the action of enzymes. This could explain that kombucha produced by fermentation with tea residue had a higher FRAP value. On the other hand, green tea residue showed a stronger effect than black tea residue, probably with the reason that green tea is unfermented, while black tea is completely fermented. Because the cells of black tea leaves were destroyed during the fermentation process for the production of black tea, the compounds in black tea are easier dissolved in water than those in green tea during the preparation of kombucha fermentation broth. This would result in a higher content of polyphenols in green tea residues than in black tea residues, and these compounds could be further dissolved in water under the action of enzymes in kombucha broth. Therefore, fermentation with tea residue could markedly increase the FRAP values of kombucha beverages, and green tea residue exhibited a stronger effect than black tea residue.

### 3.2. TEAC Values of Kombucha with or without Tea Residue

**Figure 3:**
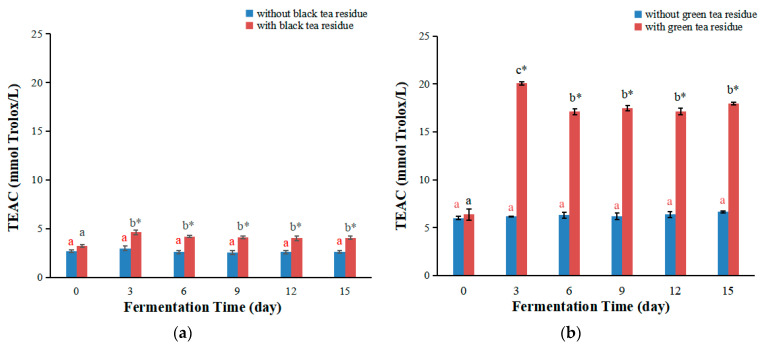
Changes of TEAC values of kombucha produced by fermentation with or without tea residue at different fermentation stages. (**a**) TEAC values of kombucha from black tea; (**b**) TEAC values of kombucha from green tea. Different letters illustrate significant differences (*p* < 0.05) for the same kombucha beverage at different fermentation times, and the same letter represents no significant difference (*p* > 0.05). Different colors of letters represent different kombucha fermentation with tea residue or without tea residue. * Indicates significant difference (*p* < 0.05) between kombucha fermentation with tea residue and kombucha fermentation without tea residue at the same fermentation time.

The antioxidant capacity of the plant beverage depends not only on its composition but also on the test system. It can be influenced by many factors and can not be fully evaluated by a single method. It is necessary to carry out more than one type of antioxidant capacity determination [29]. TEAC assay is another simple, reproducible and commonly employed method for evaluating antioxidant activity, which is to determine the ability to scavenge ABTS^∙+^ radicals [30]. Therefore, the antioxidant capacities of kombucha were also evaluated by TEAC assay, and the results are shown in Figure 3a,b.

The TEAC values of kombucha from black tea are shown in Figure 3a. Generally, the TEAC values of kombucha firstly increased with the prolonging of the fermentation time, then decreased. The maximum values were obtained on day three. The tendency of TEAC values of kombucha from black tea without tea residue was different from that reported in the literature, where the TEAC values at day 14 were higher than those at day 0 [15]. The reason could be due to the difference of the tea as well as the kombucha starter culture. The kombucha starter culture mainly contains lactic acid bacteria, acetic acid bacteria and yeast, and also contains many different microorganisms [31,32]. In addition, kombucha fermentation could also be affected by the microorganisms in the cultural environment. These microorganisms will together affect the composition of kombucha beverages. Furthermore, the TEAC values of kombucha with black tea residues were markedly higher than those without tea residues, with 1.55 and 1.61 folds higher on days three and six, respectively (Figure 3a).

The TEAC values of kombucha from green tea are shown in Figure 3b. The TEAC values of kombucha without green tea residue almost did not change during the fermentation process. The TEAC values of kombucha with green tea residue firstly increased with the increase of fermentation time, then decreased, and the maximum values were obtained on day three. The change trend of TEAC values of kombucha from green tea without tea residue was different from that reported in the literature, where the TEAC values on day 14 were higher than those on day 0 [15]. This might be associated with the differences in tea and kombucha starter cultures used in different studies. In addition, the TEAC values of kombucha with green tea residue were remarkably higher than those without tea residue, with 3.25, 2.71, 2.82, 2.69 and 2.70 folds higher on days 3, 6, 9, 12 and 15 (Figure 3b).

In short, fermentation with tea residue markedly increase the TEAC values of kombucha beverage, and green tea residue showed a stronger effect than black tea residue, which was similar to the FRAP results. These differences could be caused by many factors, similar to those of FRAP values as mentioned above.

### 3.3. TPC Values of Kombucha with or without Tea Residue

**Figure 4:**
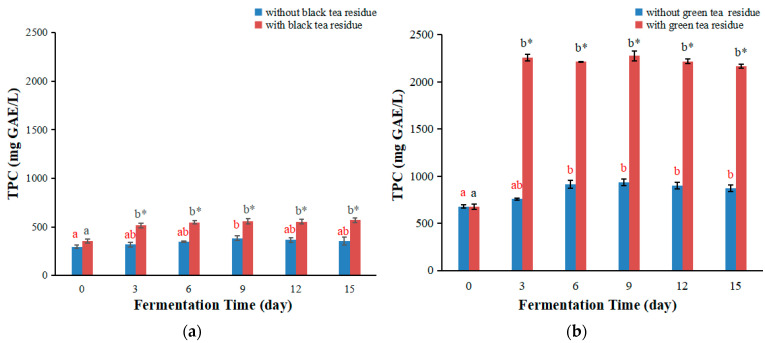
Changes of TPC values of kombucha produced by fermentation with or without tea residue at different fermentation stages. (**a**) TPC values of kombucha from black tea; (**b**) TPC values of kombucha from green tea. Different letters illustrate significant differences (*p* < 0.05) for the same kombucha beverage at different fermentation times, and the same letter represents no significant difference (*p* > 0.05). Different colors of letters represent different kombucha fermentation with tea residue or without tea residue. * Indicates significant difference (*p* < 0.05) between kombucha fermentation with tea residue and kombucha fermentation without tea residue at the same fermentation time.

The Folin-Ciocalteu method is used to determine TPC of plant samples and relies on the transfer of electrons from phenolic compounds to the Folin-Ciocalteu reagent in alkaline media. It is a simple and reproducible method and has been used in many studies [33]. The TPC values of kombucha were determined by the Folin-Ciocalteu method, and the results are shown in Figure 4.

The TPC values of kombucha from black tea are shown in Figure 4a, which firstly increased with the increasing fermentation time, then almost remained constant. The maximum values were obtained on day nine. The tendency of TPC values of kombucha from black tea without tea residue was different from that reported in the literature, where the TPC values increased with the increase of fermentation time [14,28], which could be related to the differences of tea and kombucha starter culture used in different studies. In addition, the TPC values of kombucha with black tea residue were significantly higher than those without tea residue, with 1.62, 1.58, 1.45, 1.51 and 1.60 folds higher on days 3, 6, 9, 12 and 15 (Figure 4a).

The TPC values of kombucha from green tea are shown in Figure 4b. The TPC values of kombucha without tea residue firstly rose with the extension of fermentation time, and arrived at the peak on day six, then almost maintained constant. The TPC values of kombucha with tea residue increased from day zero to day three of fermentation time, then almost kept constant. The change trend of TPC values of kombucha from green tea without tea residue was different from that reported in the literature, where the TPC values increased with the increase of fermentation time [14]. This might also be due to the differences in teas as well as the kombucha starter cultures used in different studies. Furthermore, the TPC values of kombucha with green tea residue were remarkably higher than those without tea residue, with 2.98, 2.42, 2.44, 2.46 and 2.49 folds higher on days 3, 6, 9, 12 and 15, respectively (Figure 4b).

Collectively, fermentation with tea residue markedly augmented the TPC values of kombucha beverage, and green tea residue showed a stronger effect than black tea residue, which was in accordance with the FRAP and TEAC results. These differences could be caused by many factors, which were similar to those of FRAP and TEAC values as mentioned above.

### 3.4. Contents of Phenolic Compounds and Caffeine of Kombucha with or without Tea Residue

**Figure 5:**
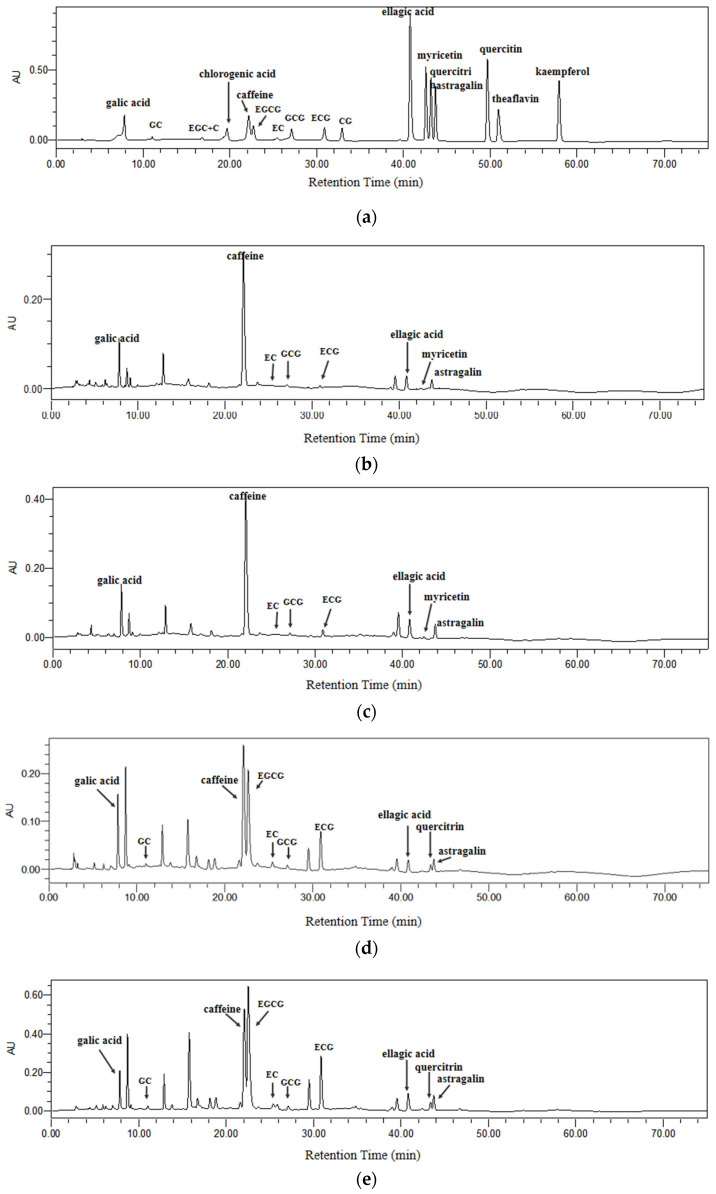
The chromatograms of kombucha produced by fermentation with or without tea residue using HPLC-PDAD at 245 nm. (**a**) standards, (**b**) kombucha from black tea without tea residue, (**c**) kombucha from black tea with tea residue, (**d**) kombucha from green tea without residue, and (**e**) kombucha from green tea with tea residue. C, catechin; CG, catechin gallate; EC, epicatechin; ECG, epicatechin gallate; EGC, epigallocatechin; EGCG, epigallocatechin gallate; GC, gallocatechin; GCG, gallocatechin gallate.

Polyphenols and caffeine in kombucha beverages were separated and identified by HPLC via comparing their retention time and ultraviolet-visible spectra with those of standard compounds, and several representative chromatograms are shown in Figure 5. Eight compounds were identified from kombucha beverages made by black tea, including galic acid, caffeine, epicatechin, gallocatechin gallate, epicatechin gallate, ellagic acid, myricetin and astragalin (Figure 5b,c). In addition, 10 compounds were identified from kombucha beverages made by green tea, including galic acid, gallocatechin, caffeine, epigallocatechin gallate, epicatechin, gallocatechin gallate, epicatechin gallate, ellagic acid, quercitrin and astragalin (Figure 5d,e). As seen from Figure 5, the chromatographic peaks of kombucha with tea residue were generally higher than those without tea residue, that is, Figure 5c vs. Figure 5b as well as Figure 5e vs. Figure 5d.

**Figure 6:**
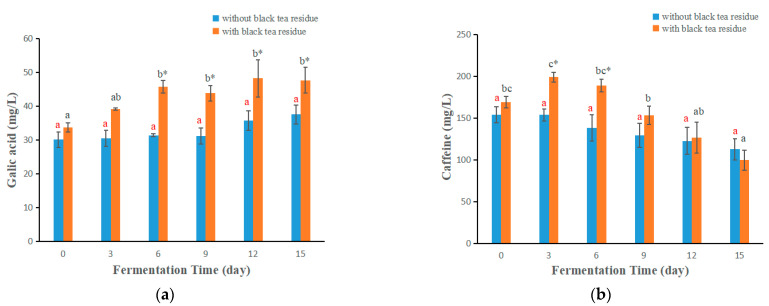

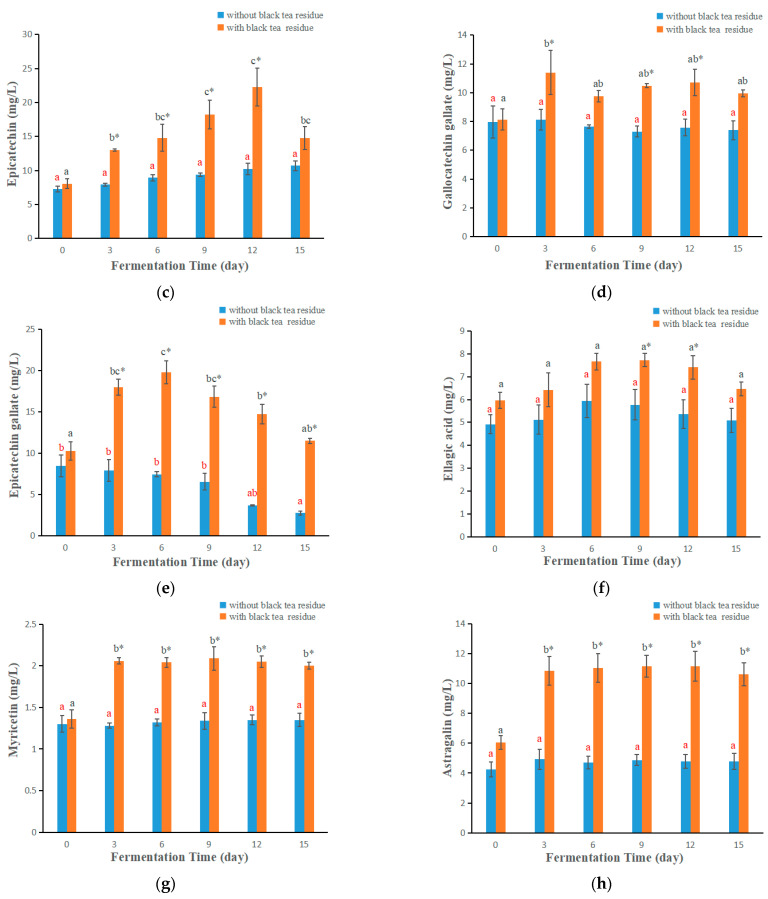

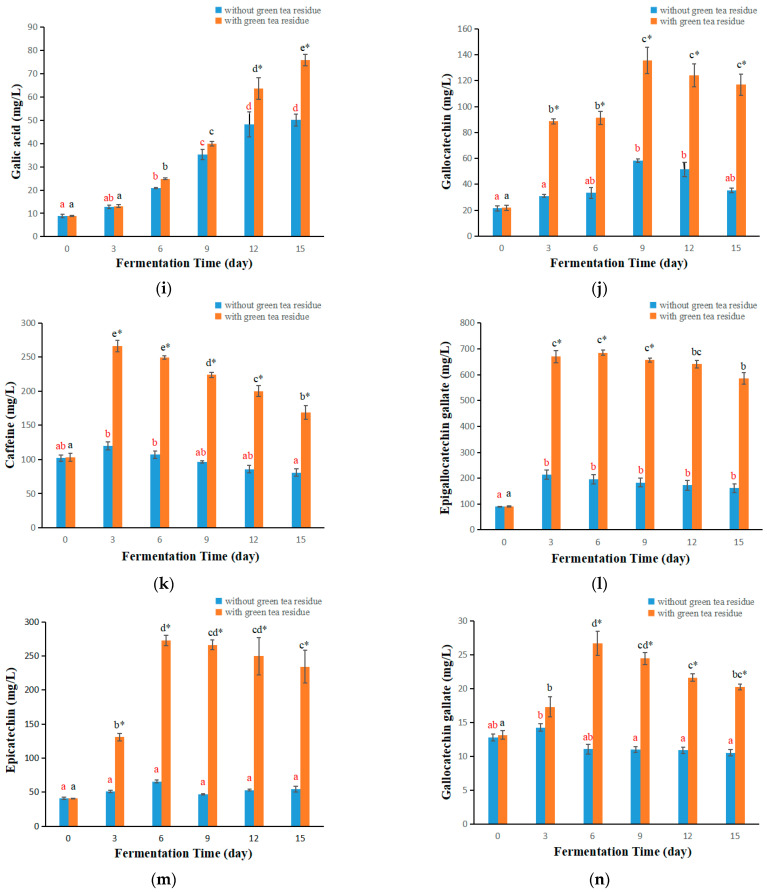

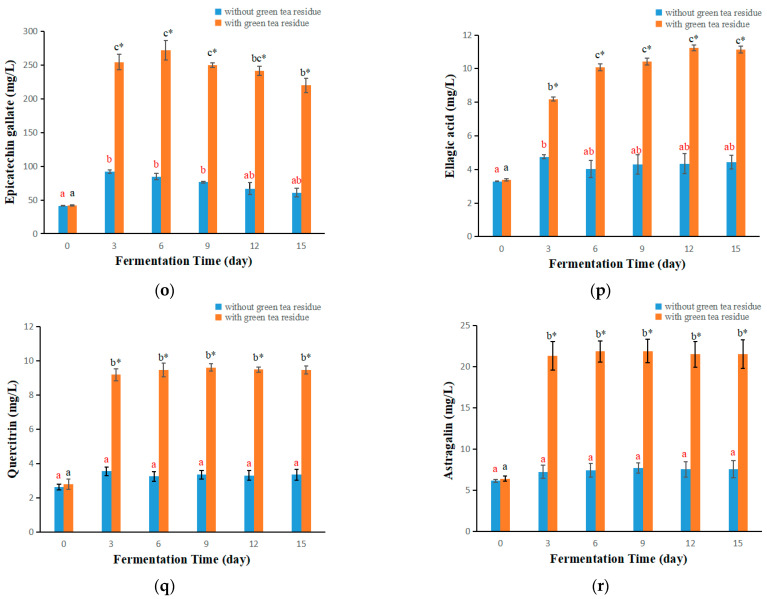
The changes of contents of main phenolic compounds and caffeine of kombucha produced by fermentation with or without black tea residue at different fermentation stages. (**a**–**h**) kombucha from black tea, and (**i**–**r**) kombucha from green tea. Different letters illustrate significant differences (*p* < 0.05) for the same kombucha beverage at different fermentation times, and the same letter represents no significant difference (*p* > 0.05). Different colors of letters represent different kombucha fermentation with tea residue or without tea residue. * Indicates significant difference (*p* < 0.05) between kombucha fermentation with tea residue and kombucha fermentation without tea residue at the same fermentation time.

The peak area under the maximal absorbance wavelength was used for the quantification of phenolic compounds and caffeine in kombucha beverages, and the results are given in Figure 6. Generally, the changes of concentrations of phenolic compounds and caffeine with the fermentation time were very different, which were similar to those reported in the literature [27,34]. In addition, the concentrations of phenolic compounds and caffeine in kombucha with tea residue were higher than those without tea residue (Figure 6). Seen from Figure 6, the change trends of these compounds mainly showed four types: (1) The concentration increased with the increase of fermentation time (e.g., gallic acid, see Figure 6i), which could be because other compounds from tea were degraded to produce it. This was in accordance with that reported in our previous paper, where the extract of green tea was enzymatically degraded with tannase, and the content of gallic acid markedly increased [35]. (2) The concentration decreased with the increase of fermentation time (e.g., caffeine in kombucha without black tea residue, see Figure 6b), which could be degraded by microorganisms. For kombucha with black tea residue (Figure 6b), the concentration of caffeine firstly increased with the increase of fermentation times, and then decreased. This was because the content of caffeine dissolved from tea residue was higher than that degraded by microorganisms during the first three days. (3) The concentration almost did not change with fermentation time (e.g., myricetin in kombucha without black tea residue, see Figure 6g). This could be because myricetin was not degraded by the microorganisms in kombucha beverages. For kombucha with black tea residue (Figure 6g), the concentration of myricetin firstly increased with the increase of fermentation time, and then almost did not change with fermentation time, which could be because myricetin continue to dissolve from tea residue during the first three days. (4) The concentration firstly increased with the increase of fermentation time, and then decreased (e.g., gallocatechin, see Figure 6j). This could be because other compounds from tea were degraded to produce it, and on the other hand, it could be degraded by the microorganisms in kombucha beverages. The gallocatechin produced from other compounds was bigger than that degraded during the first nine days, and then the produced gallocatechin was lower than that degraded after day nine. As for why some compounds were degraded and some not. Because kombucha fermentation was carried out in a clean and dark place at room temperature, chemical degradation would be minor, and biological degradation would be major. The biological degradation mainly depends on the species of microorganisms (or enzymes) in kombucha and the chemical structures of compounds. When the enzymes fit with the chemical structures of compounds, the compound would be degraded, otherwise, would not be degraded. Therefore, some compounds were degraded and some not, because of their structural differences.

In a word, fermentation with tea residue markedly increased the concentrations of some phenolic compounds and caffeine in kombucha, and green tea residue showed a stronger effect than black tea residue, which was in accordance with the FRAP, TEAC and TPC results.

### 3.5. Correlations Analysis between Parameters and Compounds

**Figure 7:**
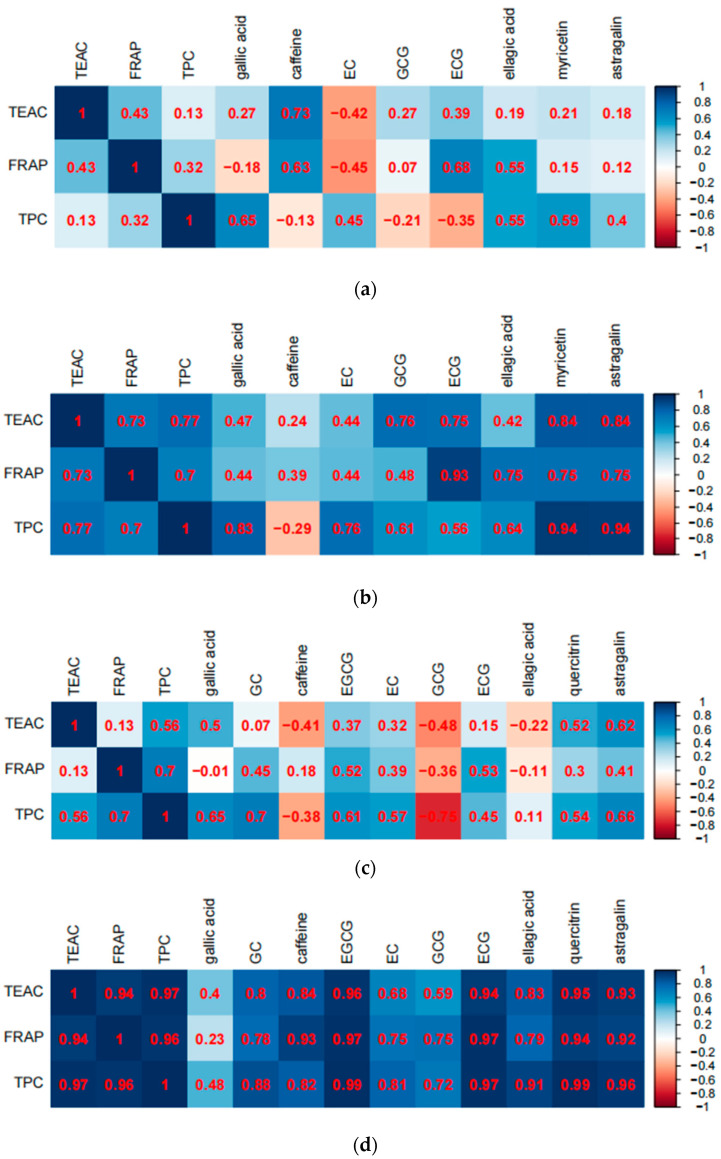
Heat maps analysis of the correlations between parameters and compounds. (**a**) kombucha from black tea without tea residue, (**b**) kombucha from black tea with tea residue, and (**c**) kombucha from green tea without residue, and (**d**) kombucha from green tea with tea residue.

The correlations between parameters and compounds have been analyzed, and the results are given in Figure 7. For TEAC vs. FRAP values, no significant correlations were observed from kombucha produced by fermentation without black tea residue (R = 0.43) or green tea residue (R = 0.13), hinting that the compounds capable of reducing oxidants (such as ferric ions) could be different from those scavenging free radicals in these kombuchas, while significant relationships were shown in kombucha produced by fermentation with black tea residue (R = 0.73) or green tea residue (R = 0.94), suggesting that antioxidant components in these kombuchas could reduce oxidants and scavenge free radicals. For FRAP vs. TPC values, no significant relationship was found in kombucha produced by fermentation without black tea residue (R = 0.32), but significant correlations were observed from kombucha produced by fermentation without green tea residue (R = 0.70) and fermentation with black tea residue (R = 0.70) or green tea residue (R = 0.96), indicating that phenolic compounds could be one of the main components responsible for reducing the ability of these kombuchas. For TEAC vs. TPC values, no significant relationships were shown in kombucha produced by fermentation without black tea residue (R = 0.13) or green tea residue (R = 0.56), but significant correlations were seen from kombucha produced by fermentation with black tea residue (R = 0.77) or green tea residue (R = 0.97), suggesting that phenolic compounds could be one of the main components responsible free radicals scavenging ability of these kombuchas.

For the relationships between FRAP values and bioactive compounds in kombucha from black tea: (1) significant correlations existed between FRAP and the following compounds in kombucha without tea residue, including caffeine (R = 0.63) and ECG (R = 0.68); and (2) significant relations were observed between FRAP and the following constituents in kombucha with tea residue, including ECG (R = 0.93), ellagic acid (R = 0.75), myricetin (R = 0.75) and astragalin (R = 0.75). For the relationships between FRAP values and bioactive compounds in kombucha from green tea: (1) no significant correlation existed between FRAP and the tested compounds in kombucha without tea residue; and (2) significant relations were observed between FRAP and the following constituents in kombucha with tea residue, including GC (R = 0.78), caffeine (R = 0.93), EGCG (R = 0.97), EC (R = 0.75), GCG (R = 0.75), ECG (R = 0.97), ellagic acid (R = 0.79), quercitrin (R = 0.94) and astragalin (R = 0.92).

For the relationships between TEAC values and bioactive compounds in kombucha from black tea: (1) significant correlations existed between TEAC and caffeine (R = 0.73) in kombucha without tea residue; and (2) significant relations were observed between TEAC and the following constituents in kombucha with tea residue, including GCG (R = 0.76), ECG (R = 0.75), myricetin (R = 0.84) and astragalin (R = 0.84). For the relationships between TEAC values and bioactive compounds in kombucha from green tea: (1) a significant correlation was found between TEAC and astragalin (R = 0.62) in kombucha without tea residue; and (2) significant relations were observed between TEAC and the following constituents in kombucha with tea residue, including GC (R = 0.80), caffeine (R = 0.84), EGCG (R = 0.96), EC (R = 0.68), ECG (R = 0.94), ellagic acid (R = 0.83), quercitrin (R = 0.95) and astragalin (R = 0.93).

For the relationships between TPC values and bioactive compounds in kombucha from black tea: (1) a significant correlation existed between TPC and gallic acid (R = 0.65) in kombucha without tea residue; and (2) significant relations were observed between TPC and the following constituents in kombucha with tea residue, including gallic acid (R = 0.83), EC (R = 0.76), GCG (R = 0.61), ellagic acid (R = 0.64), myricetin (R = 0.94) and astragalin (R = 0.94). For the relationships between TPC values and bioactive compounds in kombucha from green tea: (1) significant correlations existed between TPC and the following compounds in kombucha without tea residue, including gallic acid (R = 0.65), GC (R = 0.70), EGCG (R = 0.61) and astragalin (R = 0.66); and (2) significant relations were found between TPC and the following constituents in kombucha with tea residue, including GC (R = 0.88), caffeine (R = 0.82), EGCG (R = 0.99), EC (R = 0.81), GCG (R = 0.72), ECG (R = 0.97), ellagic acid (R = 0.91), quercitrin (R = 0.99) and astragalin (R = 0.96).

In short, more tested compounds in kombucha produced by the fermentation with tea residue contributed to FRAP, TEAC and TPC values than those in kombucha produced by the fermentation without tea residue. Furthermore, more tested compounds in kombucha with green tea residue contributed to FRAP, TEAC and TPC values than those in kombucha with black tea residue. In addition, the relationships of TEAC vs. FRAP, FRAP vs. TPC and TEAC vs. TPC were similar to the relationships of FRAP, TEAC, and TPC values with the tested compounds.

### 3.6. Sensory Analysis

**Figure 8:**
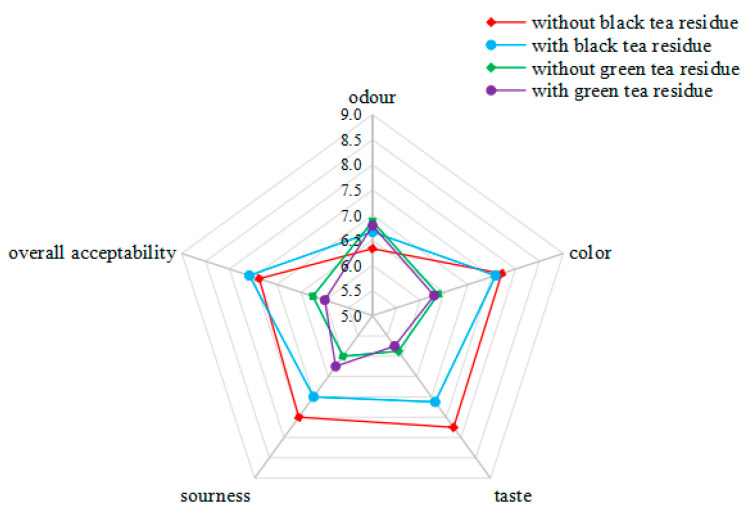
Sensory analysis.

The sensory analysis of kombucha beverages was also performed, and the results are given in Figure 8. For kombucha from black tea without tea residue or with tea residue, there were no significant differences in the scores of odour, color, taste, sourness and overall acceptability (*p* > 0.05). For kombucha from green tea without residue or with tea residue, there were also no significant differences in the scores of five evaluated sensory parameters (*p* > 0.05). In addition, there were no significant differences among the scores for odour of kombucha beverages from black tea or green tea (*p* > 0.05). However, kombucha from black tea (with or without tea residue) had higher scores than those from green tea (with or without tea residue) for the color and taste (*p* < 0.05). Furthermore, kombucha from black tea without tea residue had higher scores than kombucha from green tea (with or without tea residue) for the sourness (*p* < 0.05), and kombucha from black tea (with or without tea residue) had higher scores than kombucha from green tea with tea residue for the overall acceptability (*p* < 0.05). Generally, there were no significant differences between kombucha from black tea without tea residue and with tea residue or between kombucha from green tea without residue and with tea residue, but kombucha from black tea obtained higher scores than those from green tea. In fact, the taste of kombucha products could be also adjusted using different fruits, vegetables, flowers, spices and herbs according to the demand of consumers [36].

In the present study, the kombucha beverage is prepared with tea residue for the first time, and the research has focused on the effects of tea residue on antioxidant capacities and polyphenolic contents of kombucha. In the future, many other effects should be studied, especially, the effect on bacterial cellulose films production. As shown in Figure 1, the films in kombucha produced by the fermentation with tea residue were much thicker than those without tea residue. These films can be used as a suitable raw material in several fields such as the food industry, biomaterial preparation, fashion and textile industries, as well as environmental engineering [37,38].

## 4. Conclusions

In this study, a kombucha beverage was produced with tea residue for the first time. The results showed that fermentation with tea residue remarkably enlarged the antioxidant activity and total polyphenolic content of kombucha beverages, and green tea residue showed a stronger effect than black tea residue. In addition, 8 compounds and 10 compounds in kombucha beverages produced with black tea and green tea were identified and quantified by HPLC-PDAD, respectively, and the changes of their concentrations were quite different with the fermentation time. The changing trends of these compounds were basically in accordance with the change of FRAP, TEAC, and TPC values, which indicated that these compounds at least partly contributed to the antioxidant activity of kombucha. Furthermore, the concentrations of polyphenols and caffeine in kombucha beverages produced with tea residue were higher than those without tea residue. Therefore, fermentation with tea residue can be a better strategy to produce kombucha, and is especially feasible in domestic production.

## Data Availability

Data is contained within the article.
